# Clinical outcomes after percutaneous coronary intervention for early versus late and very late stent thrombosis: a systematic review and meta-analysis

**DOI:** 10.1007/s11239-020-02184-7

**Published:** 2020-07-20

**Authors:** Yi-Xing Yang, Yin Liu, Xiao-Wei Li, Peng-Ju Lu, Jiao Wang, Chang-Ping Li, Jing Gao

**Affiliations:** 1 Tianjin Medical University, No.22 Qi xiang tai Road, Heping District, Tianjin, 300070 People’s Republic of China; 2 Department of Cardiology, Tianjin Chest Hospital, No. 261 Tai er zhuang Road, Jinnan District, Tianjin, 300222 People’s Republic of China; 3 Cardiovascular Institute, Tianjin Chest Hospital, No.261 Tai er zhuang Road, Jinnan District, Tianjin, 300222 People’s Republic of China

**Keywords:** Stent thrombosis, Outcomes, Percutaneous coronary intervention, Meta-analysis

## Abstract

Whether the clinical outcomes of stent thrombosis (ST) are different when stratified by time of occurrence remains unclear. The objective of this study was to compare the short- and long-term clinical outcomes after percutaneous coronary intervention (PCI) for early stent thrombosis (EST) versus late stent thrombosis (LST) and very late stent thrombosis (VLST). We enrolled eligible studies searched from the main electronic databases (EMBASE, PubMed, Cochrane). The primary endpoints were in-hospital, 30-day, 1-year and long-term mortality. The secondary endpoints included recurrent stent thrombosis (RST) and target vessel/lesion revascularization (TVR/TLR) during hospitalization, at 30 days, at 1 year and at long-term follow-up. A total of 23 studies with 17,592 patients were included. Compared with mortality rates of the late and very late thrombosis (LST/VLST) group, in-hospital (P = 0.004), 30-day (P < 0.00001), 1-year (P < 0.00001) and long-term mortality rates (P = 0.04) were significantly higher in the EST group. The in-hospital TVR/TLR rates were similar between the EST group and the LST/VLST group. However, a higher trend in TVR/TLR rate at 30 days and a significantly higher TVR/TLR rate at 1 year (P = 0.002) as well as at long-term follow up (P = 0.009) were found in the EST group. EST patients also trended toward higher risk of RST in both short- and long-term follow-up than LST/VLST patients, although differences were not statistically significant. After PCI treatment, patients with EST have worse clinical outcomes in both short- and long-term follow-up than patients with LST/VLST. Further studies are warranted to determine the optimal treatment strategies for EST.

**Electronic supplementary material:**

The online version of this article (10.1007/s11239-020-02184-7) contains supplementary material, which is available to authorized users.

## Highlights

- This is the first meta-analysis to investigate the associations between the timing of ST occurrence and the clinical outcomes of ST.
- Patients with EST have worse clinical outcomes in both short- and long-term follow-up than patients with LST/VLST.
- Further studies are warranted to determine the optimal treatment strategies for EST.

## Introduction

Stent thrombosis (ST) is a rare but catastrophic complication of PCI with high mortality in both short-term and long-term periods [1, 2]. According to the Academic Research Consortium criteria, ST can be stratified into early stent thrombosis (EST), occurring within 30 days after index PCI, late stent thrombosis (LST), occurring from 30 days to 1 year after index PCI, and very late stent thrombosis (VLST), occurring more than 1 year after index PCI [3]. Recently, several studies investigated the associations between the timing of ST occurrence and the clinical outcomes of ST, but the results were inconsistent [4–26]. Therefore, we conducted a meta-analysis to compare the short-term and long-term clinical outcomes following PCI for patients with EST versus patients with LST and VLST.

## Methods

A study protocol was developed prior to data collection and was registered on PROSPERO and can be accessed at https://www.crd.york.ac.uk/prospero/display_record.php?ID=CRD42019144994.

### Search strategy and study selection

We searched the literature in the PUBMED, EMBASE and Cochrane Library databases, using combinations of the following key words: “outcome” OR “prognosis” AND “early stent thrombosis” OR “acute stent thrombosis” OR “subacute stent thrombosis” OR “late stent thrombosis.” An initial screen of titles and abstracts was conducted to exclude studies that were irrelevant to the present study. Full-text of the relevant articles were evaluated by the selection criteria. Studies were eligible for inclusion if they: (1) compared the clinical outcomes of EST versus LST or VLST; (2) had angiographically confirmed (definitive) ST; (3) included PCI treatment for ST; (4) follow-up time including in-hospital, 30-day, 1-year and long-term periods (> 1 year); (5) had at least 30 participants; and (6) were randomized clinical trials, observational studies or abstracts with sufficient data. Studies were excluded if they: (1) did not compare the clinical outcomes of EST versus LST or VLST; (2) included probable or possible ST; (3) included unclear treatment for ST; (4) had other follow-up periods such as 7-day, 180-day, etc.; (5) participants were fewer than 30; and (6) were categorized as case reports or comments. In addition, reference lists of the selected studies were also screened for potential articles.

### Data extraction and quality assessment

Data extraction was performed using a standardized data collection form. The primary endpoints were in-hospital, 30-day, 1-year and long-term mortality. The secondary endpoints included RST and TVR/TLR during hospitalization, at 30 days, at 1 year and at long-term follow-up. Definitions of “ST”, “RST”, “TVR” and “TLR” corresponded with the Academic Research Consortium criteria [3]. Study quality was assessed by using the Newcastle–Ottawa Scale [27]. Two reviewers independently performed the study search and selection, data extraction and quality assessment of the selected studies. Disagreements were resolved by team discussion.

### Statistical analysis

Results were analyzed using computed pooled risk ratios (RR) with 95% confidence intervals (CIs). Statistical heterogeneity was evaluated by the Cochrane Q test and the I^2^ statistic. A random-effect model was used when a significant heterogeneity (P < 0.05 or I^2^ > 50) was detected, otherwise, a fixed-effect model was used (P ≥ 0.05 or I^2^ ≤ 50%). To analyze intuitively, LST and VLST patients were combined as the control group for EST. Statistical analysis was carried out using the REVIEW MANAGER software (Version 5.3, Cochrane Collaborative, Oxford, England).

## Results

### Study characteristics

**Fig. 1:**
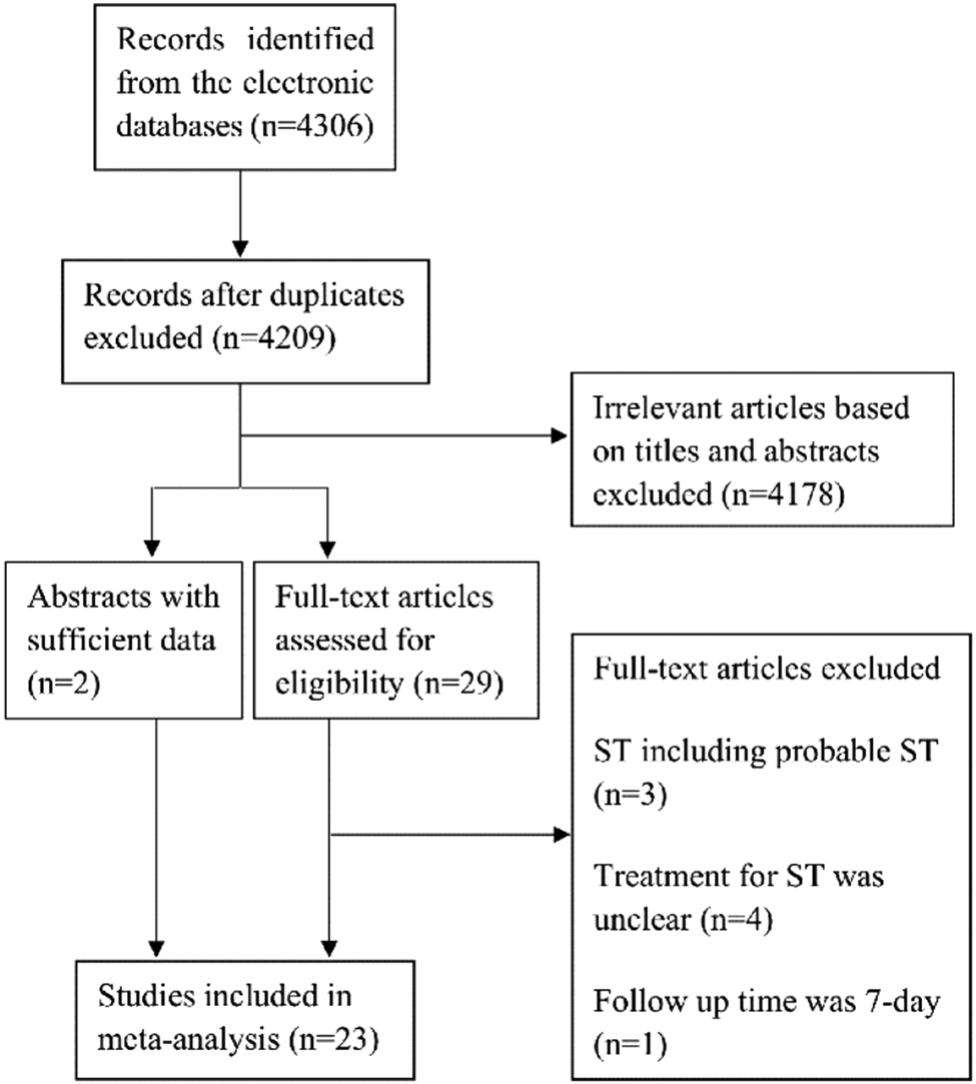
Flow diagram of literature search strategy process

The literature search strategy process is shown in Fig. 1. From the 4306 published studies identified, 21 observational studies and 2 abstracts with a total of 17,592 patients were finally enrolled in our analyses. Among the patients enrolled, 4937 patients had EST, and 12,655 patients had LST/VLST. The main characteristics of the included studies are shown in Table 1. Quality assessment of the studies is shown in Supplementary Table 1.

**Table 1:** Main characteristics of the included studies

Author	Year	Study type	PN	Initial stent type	Type of ST	Manifestation of ST	Treatment for ST	Follow up time and endpoints
Lemesle [4]	2009	Observational	91	DES	Definite	STEMI 74.7%	PCI	IH/1 M/1Y death, RST, MI, MACE
Margolis [5]	2016	Observational	83	NA	Definite	STEMI 100%	PCI	1 M death
Jones [6]	2013	Observational	194	DES/BMS	definite	STEMI 100%	PCI	LT MACE
Kimura [7]	2010	Observational	611	DES	Definite	STEMI 69.0%	PCI	1 M/1Y/LT death
Kubo [8]	2014	Observational	152	DES/BMS	Definite	AMI 81.6%	PCI	IH/1Y/LT death; 1Y/LT TLR, CD, MACE; LT RST
Armstrong [9]	2012	Observational	7079	DES/BMS	Definite	STEMI 64.2% (AMI 87.1%)	PCI	IH death
de la TH [10]	2008	Observational	301	DES	Definite	STEMI 83.7%	PCI	IH/LT death, RST
Daemen [11]	2007	Observational	152	DES	Definite	AMI 45.4%	PCI	IH/1 M death, RST, TVR
Singh [12]	2018	Observational	46	DES/BMS	Definite	STEMI 82.6%	PCI	IH/LT death
Kuramitsu [13]	2019	Observational	313	DES	Definite	NA	PCI	1 M/1Y/LT death, RST
Mahmoud [14]	2011	Observational	113	DES/BMS	Definite	STEMI 85.0% (AMI 100%)	PCI	1 M/1Y death
Lempereur [15]	2016	Observational	101	DES/BMS	Definite	STEMI 62.5%	PCI	1 M/1Y death, TVR, MACE
Kim [16]	2019	Observational	243	DES/BMS	Definite	STEMI 63.8% (AMI 89.7%)	PCI	1Y MACE
Armstrong [17]	2014	Observational	656	NA	Definite	NA	PCI	1 M death
Almalla [18]	2013	Observational	106	DES/BMS	Definite	STEMI 78.3%	PCI	LT MACE
Van Werkum [19]	2009	Observational	431	DES/BMS	Definite	NA	PCI	LT MACE
Katsikis [20]	2019	Observational	131	DES/BMS	Definite	STEMI 88.0%	PCI	LT death
Yeo [21]	2015	Observational	210	DES/BMS	Definite	STEMI 65.0% (AMI 90.0%)	PCI	LT MACE
Konishi [22]	2019	Observational	370	DES	Definite	AMI 29.5%	PCI	IH death
Tovar Forero [23]	2019	Observational	679	DES/BMS	Definite	AMI 87.2%	PCI	LT MACE
Feldman [24]	2011	Abstract	5319	DES/BMS	Definite	STEMI 62.2% (AMI 84.8%)	PCI	IH death
Shimotakahara [25]	2013	Abstract	102	BMS	Definite	NA	PCI	LT death, TLR
Kukreja [26]	2009	Observational	109	DES/BMS	Definite	NA	PCI	LT death

*PN* patient number, *ST* stent thrombosis, *DES* drug-eluting stent, *BMS* bare-metal stent, *NA* not available, *IH* in-hospital, *PCI* percutaneous coronary intervention, *1 M* 1 month, *1Y* 1 year, *LT* long-term, *MACE* major adverse cardiovascular event, *RST* recurrent stent thrombosis, *AMI* acute myocardial infarction, *STEMI* ST segment elevation myocardial infarction, *TVR* target vessel revascularization, *TLR* target lesion revascularization, *CD* cardiac death, *MI* myocardial infarction

### Patients’ characteristics

Table 2 shows the baseline clinical characteristics of patients in the two groups. Compared with those with LST/VLST, patients with EST were more frequently diabetics and presented with cardiogenic shock (CS) at the time of ST (diabetics: 41.6% vs. 31.3%, P = 0.0004, 13 studies including 15,905 patients were used for this analysis, Supplemental Fig. 1a); (CS: 13.7% vs. 8.9%, P < 0.00001, 10 studies including 14,181 patients contributed to this analysis, Supplemental Fig. 1b). However, male gender and hyperlipemia were more frequent in patients with LST/VLST than in those with EST (male: 77.0% vs. 72.1%, P = 0.03, 13 studies including 15,907 patients were used for this analysis, Supplemental Fig. 2a); (hyperlipemia: 85.7% vs. 71.4%, P < 0.00001, 9 studies including 9217 patients contributed to this analysis, Supplemental Fig. 2b). Four studies including 8188 patients reported that chronic kidney disease (CKD) was higher in the EST group than in the LST/VLST group (4.6% vs. 2.3%, P < 0.00001, Supplemental Fig. 3a), while another seven studies including 2088 patients reported the incidence of CKD was markedly higher in the LST/VLST group than in the EST group (21.5% vs. 8.7%, P = 0.001, Supplemental Fig. 3b).

**Table 2:** Baseline clinical characteristics of patients

Study	PN		MA (years)		Male (%)		HTN (%)		DM (%)		HLP (%)		CS (%)		CKD (%)	
	EST	LST	EST	LST	EST	LST	EST	LST	EST	LST	ETS	LST	EST	LST	EST	LST
Lemesle [4]	51	40	61.4	63.5	51.0	70.0	86.3	82.5	54.9	47.5	88.2	95.0	39.2	20	21.6	22.5
Margolis [5]	35	48	66.9	65.2	83.0	87.0	71.0	73.0	37.0	31.0	71.0	87.0	NA*		NA*	
Jones [6]	67	127	62.6*		71.0*		57.5*		26.0*		55.0*		6.2*		NA*	
Kimura [7]	322	289	67.1	64.8	81.0	83.0	72.0	75.0	43.0	40.0	NA*		11.0	5.9	6.8	19
Kubo [8]	55	97	65.9	67.2	80.0	85.6	74.6	62.9	49.1	37.1	50.9	57.7	12.7	4.1	9.1	25.8
Armstrong [9]	1391	5688	61.0	60.5	67.1	75.7	85.8	85.2	43.7	30.2	85.6	89.1	13.4	9.6	3.6	2.0
de la TH [10]	149	152	62.5	58.4	60.0	78.3	53.0	45.4	40.0	21.7	43.0	53.3	NA*		7.4	2.6
Daemen [11]	91	61	61.9	58.0	73.0	80.0	39.0	46.0	28.0	8.0	45.0	54.0	9.0	8.0	9.0	2.0
Singh [12]	38	8	58.6*		73.9*		45.7*		45.7*		32.6*		18.4	12.5	NA*	
Kuramitsu [13]	179	134	68.2	67.9	84.4	74.6	78.8	79.9	45.8	50.7	81.6	82.8	8.9	2.7	3.9	6.0
Mahmoud [14]	59	54	63.5*		77.0*		44.2*		13.3*		43.4*		NA*		NA*	
Lempereur [15]	36	65	64.4	64.0	72.2	78.5	58.3	66.2	44.4	32.3	NA*		19.4	9.2	0.0	1.5
Kim [16]	110	133	64.4*		69.5*		59.7*		40.7*		9.1*		NA*		38.7*	
Armstrong [17]	129	527	65.6	64.5	98.4	99.4	NA*		53.5	50.7	NA*		NA*		9.3	4.7
Almalla [18]	86	20	69.7*		80.2*		71.6*		30.2*		NA*		28.3*		20.8*	
Van Werkum [19]	317	114	61.1*		74.9*		46.9*		23.2*		53.1*		NA*		16.9*	
Katsikis [20]	14	117	65.0*		85.0*		66.0*		23.0*		64.0*		10.0*		17.0*	
Yeo [21]	69	141	61.0*		86.0*		76.0*		39.0*		NA*		21.0*		15.0*	
Konishi [22]	287	83	68.5	69.2	80.4	81.3	51.1	65.1	40.2	41.0	41.2	48.2	NA		2.7	22.9
Tovar Forero [23]	345	334	64.1	61.4	74.2	79.9	51.2	54.8	23.6	20.7	54.1	66.2	16.9	6.9	17.8	21.5
Feldman [24]	1012	4307	62.0	60.8	68.0	74.9	NA*		43.1	30.2	NA*		13.5	8.6	NA*	
Shimotakahara [25]	40	62	NA*		NA*		NA*		NA*		NA*		NA*		NA*	
Kukreja [26]	55	54	61.8*		72.2*		38.1*		15.4*		48.5*		NA*		NA*	

*PN* patient number, *MA* mean age, *HTN* hypertension, *DM* diabetes mellitus, *HLP* hyperlipemia, *CS* cardiogenic shock (at the time of ST), *CKD* chronic kidney disease, *EST* early stent thrombosis, *LST* late stent thrombosis (including very late stent thrombosis here), *NA* not available

*Overall

**Fig. 2:**
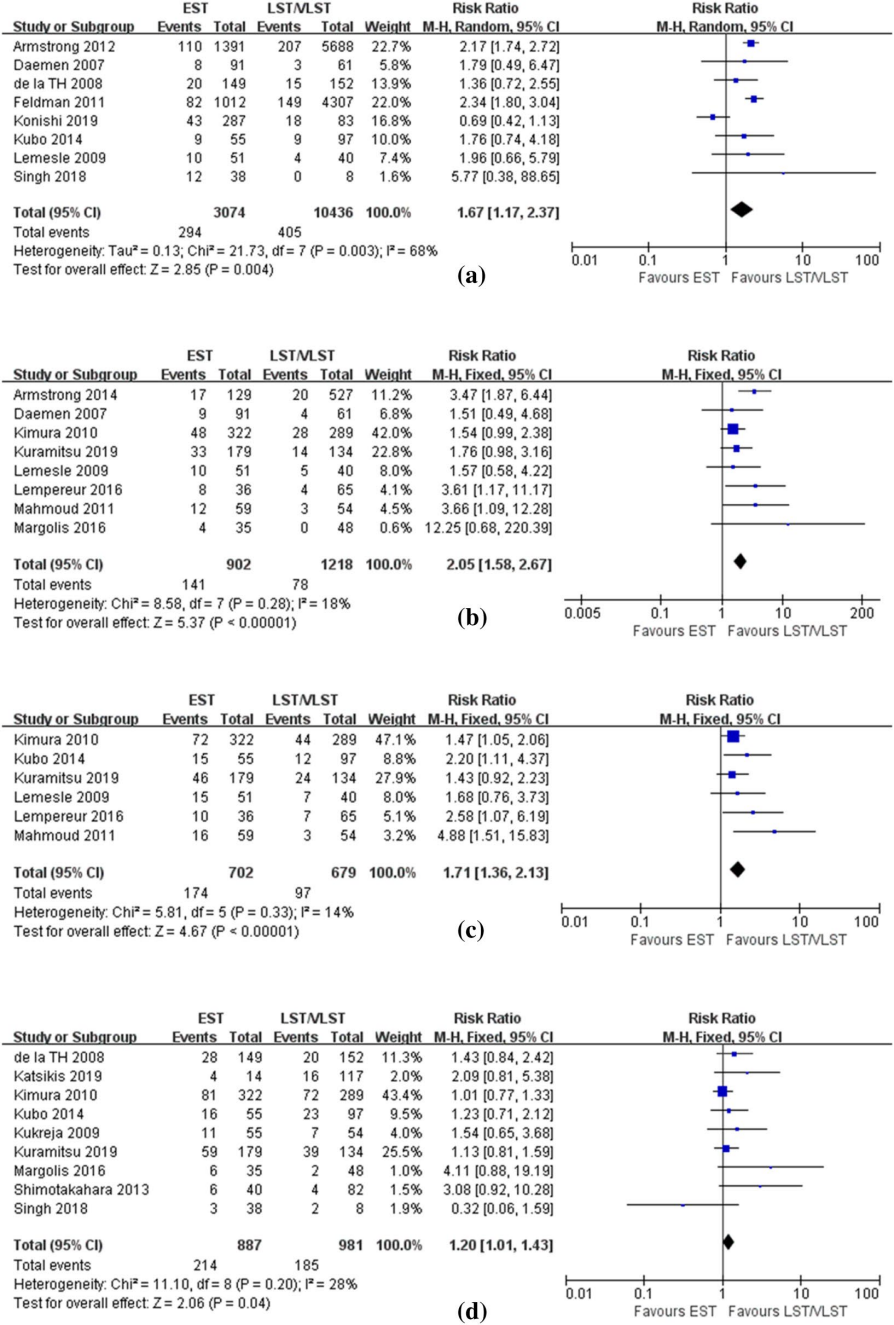
Forest plot with RR for EST vs LST/VLST (**a**) in-hospital mortality (**b**) 30-day mortality (**c**) 1-year mortality (**d**) long-term mortality

**Fig. 3:**
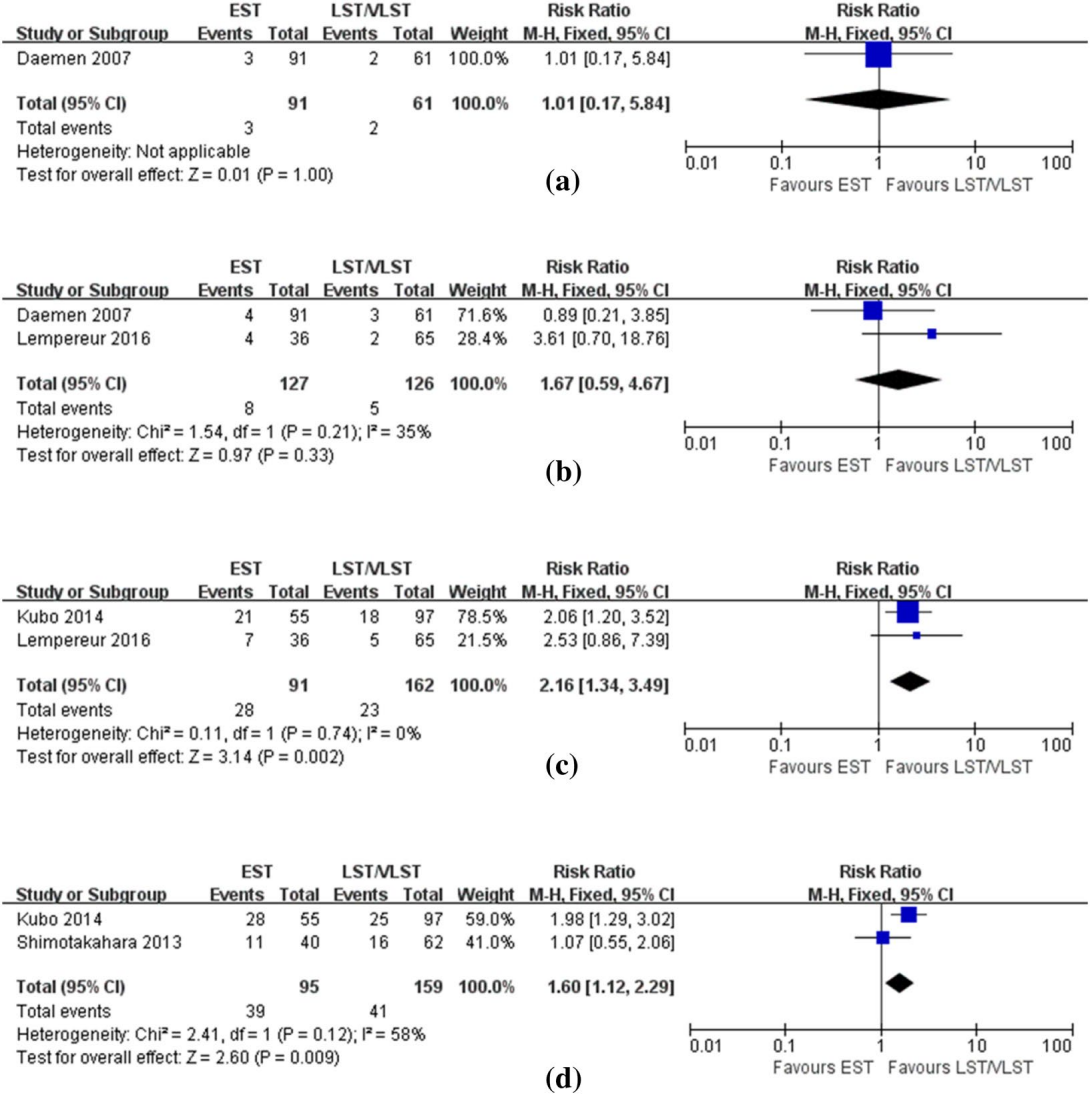
Forest plot with RR for EST vs LST/VLST (**a**) in-hospital TVR (**b**) 30-day TVR (**c**) 1-year TVR/TLR (**d**) long-term TLR

Table 3 shows the lesion and treatment features of the two groups. Compared with the LST/VLST group, the EST group had a higher rate of bifurcation lesions and left anterior descending artery (LAD) lesions (bifurcation: 23.5% vs. 15.2%, P < 0.00001, 10 studies including 10,272 patients were used for this analysis, Supplemental Fig. 4a); (LAD: 50.8% vs. 41.2%, P < 0.00001, 11 studies including 10,523 patients contributed to this analysis, Supplemental Fig. 4b). Additional stent (AS) was utilized more frequently in the LST/VLST group than in the EST group (66.0% vs. 46.8%, P < 0.00001, 13 studies including 15,530 patients were used for this analysis, Supplemental Fig. 5a), whereas intra-aortic balloon pump (IABP) and glycoprotein IIb/IIIa inhibitor (GPI) were administered more frequently in the EST group than in the LST/VLST group (IABP: 17.3% vs. 9.5%, P < 0.0001, 7 studies including 9360 patients were used for this analysis, Supplemental Fig. 6a); (GPI: 70.2% vs. 65.5%, P = 0.02, 6 studies including 8333 patients contributed to this analysis, Supplemental Fig. 6b). No significant differences were found between the two groups in the rate of using thrombus aspiration (TA, LST/VLST 34.7% vs. EST 30.4%, P = 0.37, 11 studies including 14,945 patients were used for this analysis, Supplemental Fig. 5b). The rate of achieving thrombolysis in myocardial infarction (TIMI) grade 3 post-PCI was significantly lower in the EST group than in the LST/VLST group (88.3% vs. 92.6%, P < 0.00001, 8 studies including 11,483 patients were used for this analysis, Supplemental Fig. 7).

**Table 3:** Lesion and treatment characteristics of patients

Study	AS (%)		TA (%)		GPI (%)		IABP (%)		LAD (%)		Bifurcation (%)		Post TIMI 3 (%)	
	EST	LST	EST	LST	EST	LST	EST	LST	EST	LST	EST	LST	EST	LST
Lemesle [4]	58.9	60	43.1	35	52.9	41.0	28.0	10.0	51.0	42.5	NA*		NA*	
Margolis [5]	NA*		NA*		NA*		NA*		NA*		NA*		NA*	
Jones [6]	86.5*		51.5*		89.0*		NA*		56.2*		NA*		NA*	
Kimura [7]	32.0	40.1	78.0	76.6	NA*		41.0	28.1	56.0	56.0	29.0	25.0	84.0	84.0
Kubo [8]	38.7	53.6	67.2	72.2	NA*		25.5	14.4	45.2	47.5	62.9	37.4	88.7	95.0
Armstrong [9]	51.2	66.5	32.1	33.0	73.9	67.2	13.4	9.2	48.0	38.9	17.3	14.2	91.6	94.4
de la TH [10]	48.0	50.0	30.0	47.8	68.0	63.7	NA*		72.0	74.3	6.0	2.6	87.0	81.1
Daemen [11]	33.0	48.0	12.0	12.0	39.0	30.0	NA*		54.0	54.0	36.0	13.0	NA*	
Singh [12]	15.6	25.0	NA*		89.2	88.9	NA*		63.0*		2.2*		60.5	100
Kuramitsu [13]	NA*		NA*		NA*		NA*		38.6	26.1	46.4	35.8	NA*	
Mahmoud [14]	62.8*		49.0	51.0	77.0*		17.7*		51.3*		40.7		NA*	
Lempereur [15]	63.9	61.5	45.7	38.5	NA*		27.8	3.1	55.6	33.8	44.4	32.3	90.9	84.6
Kim [16]	10.7*		23.0*		30.0*		8.2*		49.6*		48.1*		NA*	
Armstrong [17]	52.7	68.1	NA*		NA*		13.2	4.0	40.5	33.4	6.8	7.3	85.8	86.4
Almalla 15]	68.9*		15.1*		83.0*		NA*		62.3*		NA*		NA*	
Van Werkum [19]	49.7*		12.8*		81.7*		NA*		62.4*		51.7*		NA*	
Katsikis [20]	50.0	65.0	57.3*		56.5*		NA*		43.5*		3.1*		NA*	
Yeo [21]	64.0*		58.0*		75.0*		16.0*		48.0*		14.0*		NA*	
Konishi [22]	25.7	29.3	66.5	56.1	NA*		NA		50.7	40.2	35.1	28.9	NA*	
Tovar Forero [23]	48.0	70.4	44.3	50.6	66.6	47.9	8.5	6.4	58.6	48.5	22.6	18.6	86.1	91.5
Feldman [24]	51.0	66.5	31.2	31.5	NA*		NA*		NA*		NA*		87.9	91.3
Shimotakahara [25]	68.6*		NA*		NA*		NA*		NA*		NA*		NA*	
Kukreja [26]	NA*		NA*		NA*		NA*		NA*		NA*		NA*	

*AS* additional stent, *TA* thrombus aspiration, *GPI* glycoprotein IIb/IIIa inhibitor, *IABP* intra-aortic balloon pump, *ST* stent thrombosis, *EST* early stent thrombosis, *LST* late stent thrombosis (including very late stent thrombosis here), *LAD* left anterior descending artery, *TIMI* thrombolysis in myocardial infarction, *NA* not available

*Overall

**Fig. 4:**
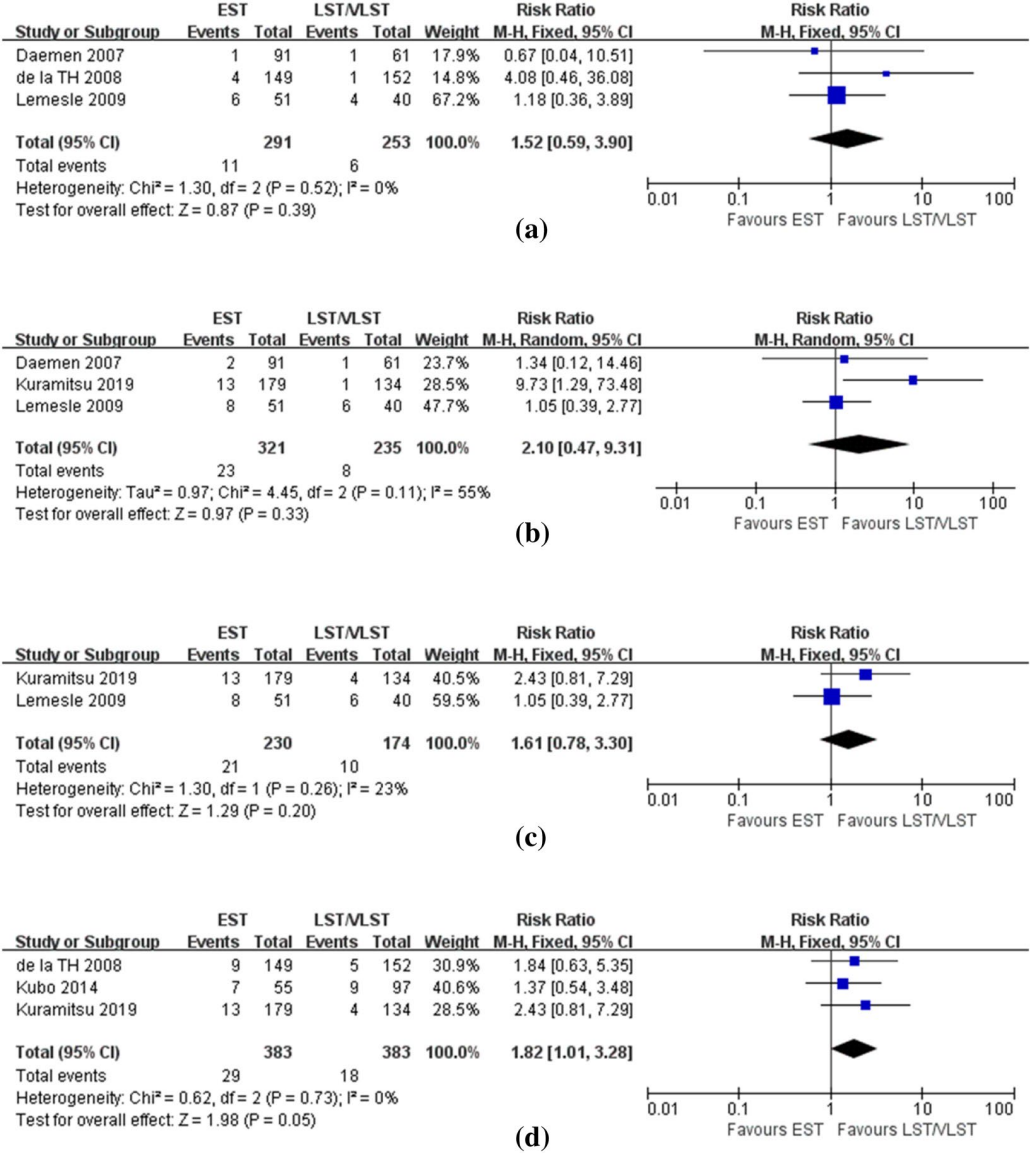
Forest plot with RR for EST vs LST/VLST (**a**) in-hospital RST (**b**) 30-day RST (**c**) 1-year RST (**d**) long-term RST

### Primary endpoints

Analysis of 8 studies including 13,510 patients demonstrated that in-hospital mortality was dramatically higher in the EST group than in the LST/VSLT group (RR: 1.67, 95% CI 1.17–2.37, P = 0.004, Fig. 2a). Analysis of 8 studies involving 2120 patients showed that 30-day mortality was significantly higher in the EST group than in the LST/VLST group (RR: 2.05, 95% CI 1.58–2.67, P < 0.00001, Fig. 2b). Moreover, 6 studies with 1381 patients contributed to the analysis of the overall mortality at 1 year, and results showed that mortality was markedly higher in the EST group than in the LST/VLST group (RR: 1.71, 95% CI 1.36–2.13, P < 0.00001, Fig. 2c). Nine studies involving 1868 patients contributed to the analysis of the overall mortality at long-term follow-up, and results demonstrated that mortality was higher in the EST group than in the LST/VLST group (RR: 1.20, 95% CI 1.01–1.43, P = 0.04, Fig. 2d).

### Secondary endpoints

Regarding TVR/TLR, only one included study with 152 patients reported the incidence of TVR during hospitalization, which was similar between the EST and LST/VLST groups (EST 3.3% vs. LST/VLST 3.28%, P = 1.00, Fig. 3a); 2 studies comprising 253 patients were used for analysis of 30-day TVR, and results showed that patients with EST had a trend toward higher risk of TVR at 30 days than patients with LST/VLST (6.3% vs. 4.0%, P = 0.33, Fig. 3b); 2 studies including 253 patients contributed to the analysis of the 1-year TVR/TLR, and results showed that the EST group had a significantly higher event rate than the LST/VLST group (30.8% vs. 14.2%, P = 0.002, Fig. 3c); 2 studies including 254 patients reported the incidence of TLR at long-term follow-up, which was also significantly higher in the EST group compared with the LST/VLST group (40.1% vs. 25.8%, P = 0.009, Fig. 3d).

In terms of RST, 3 studies with 544 patients, 3 studies with 556 patients, 2 studies with 404 patients, and 3 studies with 766 patients contributed to the analysis of the overall incidence of RST during hospitalization, at 30 days, at 1 year and at long-term follow-up, respectively. The results showed that patients with EST had a trend toward higher risk of RST during hospitalization (3.8% vs. 2.4%, P = 0.39, Fig. 4a), at 30 days (7.2% vs. 3.4%, P = 0.33, Fig. 4b), at 1 year (9.1% vs. 5.7%, P = 0.20, Fig. 4c) and at long-term follow-up (7.6% vs. 4.7%, P = 0.05, Fig. 4d), although differences were not statistically significant.

### Other outcomes of interest

One study including 152 patients reported the incidence of cardiac death (CD) at 1 year and at long-term follow-up, which were both numerically higher in the EST group than in the LST/VLST group (1-year: 23.6% vs. 11.3%, P = 0.05; long-term: 25.5% vs. 18.6%, P = 0.31). One study including 91 patients reported the rates of myocardial infarction (MI) during hospitalization, at 30 days and at 1 year, which were both numerically higher in the EST group than in the LST/VLST group (in-hospital: 27.5% vs. 20.0%, P = 0.42; 30-day: 31.4% vs. 25.0%, P = 0.31; 1 year: 37.2% vs. 35.0%, P = 0.82). Eight studies reported the incidence of major adverse cardiovascular events (MACE, defined as the combined endpoints of various outcomes), which were also higher in the EST group than in the LST/VLST group (Supplemental Table 2).

## Discussion

To the best of our knowledge, this is the first meta-analysis to investigate the associations between the timing of ST occurrence and the clinical outcomes of ST. Results showed that patients with EST had worse clinical outcomes than patients with LST/VLST in both short- and long-term follow-up after PCI treatment.

The poor clinical outcomes in EST patients were consistent with the poor angiographic outcomes in this post-PCI entity. In the present study, the rate of achievement of post-PCI TIMI flow grade 3 was significantly lower in the EST group than in the LST/VLST group. Additionally, several studies that performed quantitative coronary angiographic analysis found that patients with EST had a smaller minimum luminal diameter and a higher percentage of diameter stenosis at the end of procedure as well as at long-term angiographic follow-up than those with LST/VLST [4, 8, 11, 13].

These findings can possibly be explained as follows. First, previous studies have demonstrated that patients who develop EST are usually those with adverse baseline characteristics such as DM, STEMI, CS and multivessel diseases [1, 28]. Similarly, the present study found that patients with EST had a higher rate of DM, bifurcation lesions and LAD lesions than those with LST/VLST. This high baseline risk profile in EST patients may explain per se the poor efficacy of PCI and the higher rate of unfavorable outcomes in this entity [18, 19, 21, 23]. Moreover, the clinical presentation at time of ST was also more disastrous in EST patients than it was in LST/VLST patients. As observed in the present study, the rates of CS and IABP use at the time of presentation were higher in the EST group than in the LST/VLST group. This finding could partly explain the higher mortality in EST patients, because CS has been shown to be associated with in-hospital mortality as high as 48% and 1-year mortality as high as 58% despite aggressive treatment therapies [29].

Second, the present study also found that in patients with EST, surgeons tended to restore vessel patency by balloon angioplasty only, whereas in patients with LST/VLST, they preferred to utilize a new stent. This finding was in line with the assumption that more stent deployment-related issues may be noted in EST patients. Previous studies using intravascular imaging have identified stent underexpansion and acute malapposition (occurring during index procedure) as the most prevalent abnormalities in patients with EST. Whereas, late malapposition (occurring during follow-up), delayed endothelialization (manifesting as uncovered struts) and neoatherosclerosis have been regarded as the most important mechanisms for LST/VLST [30–33]. Moreover, the higher rates of utilizing GPI among patients with EST suggests that a higher thrombus burden may be present in this critically ill subgroup [34, 35]. Therefore, patients with EST were more likely to face more difficult and complex challenges during PCI, which may further lead to poor outcomes.

Finally, the higher adverse events rate in patients with EST may be related in part to damaged coronary collaterals. Indeed, collaterals can minimize injury to the myocardium at the time of the event and result in better outcomes [36, 37]. In patients with LST/VLST, the thrombus formation was more like a progressive evolution, thus, there was enough time for collateral circulation to develop. However, in patients with EST, the ability of establishing coronary collateral circulation may be impaired by the rapid onset of stent thrombosis due to the higher on-treatment platelet reactivity [38], which may lead to a larger myocardium infarct size and higher rates of adverse events.

Treatment of EST appears to be more challenging than that of LST/VLST, and no specific guidelines exist for optimal strategies for addressing EST. A two-step approach may be more suitable for EST. The study of Carrick et al. [39] demonstrated that, in high-risk STEMI patients, deferred stenting is associated with fewer intraprocedural thrombotic events, higher TIMI flow grade and increased myocardial salvage compared with immediate stenting. Similarly, a recent meta-analysis including 744 patients demonstrated that a deferred stent implantation strategy was associated with improved TIMI flow grade, greater TIMI myocardial blush grade and decreased MACEs without increasing major bleeding events in STEMI patients with a high thrombus burden [40]. Besides, it has been suggested that use of intravascular imaging including intravascular ultrasound (IVUS) and optical coherence tomography (OCT), which ascertains the predisposing mechanical factors of ST, may be a potential adjunctive therapy for EST [41, 42].Thus, it seems reasonable to consider that aggressive EST cases can benefit from a deferred PCI strategy with intra-coronary imaging after optimal medical therapy. Further studies are required to evaluate this speculative approach.

In the present study, heterogeneity was either low or moderate in the results of primary and secondary endpoints, but a high degree of heterogeneity was noted in the analysis of in-hospital mortality (I^2^ = 68%). Differences in patients’ clinical manifestations between the study of Konishi et al. [22] and others may account for this heterogeneity. Patients presenting with AMI at the time of ST only accounted for 29.5% in the Konishi study, whereas it accounted for 70%-90% in the other studies (Table 1). When removing the Konishi study, the heterogeneity disappeared (I^2^ = 0%) and the results became more significant (P < 0.00001, Supplemental Fig. 8).

A discrepancy regarding CKD incidence was found between studies in the EST and LST/VLST group. A possible explanation is that a significant difference exists in the incidence of CKD between patients with VLST and patients with LST (the rate was lower in VLST than in LST) [7–9, 13, 15, 17], which may lead to a certain level of bias when calculating the overall rate of CKD for the combined LST/VLST group.

Finally, male gender and dyslipidemia were found to be more frequent in patients with LST/VLST, but no dramatic evidence was found of the association between gender or dyslipidemia and the prognosis of ST, except for one study that found male gender was associated with MI at long-term follow-up [23], and another study that identified dyslipidemia as an independent predictor of composite CD and MI at five years after PCI [8].

## Limitations

First, this meta-analysis shared the limitations of the original studies. Second, the results of TVR/TLR and RST needed to be interpreted with care since the analysis might be too small to properly detect statistical differences between the two groups. Third, definitions of MACE in the individual studies were significantly different and there were limited studies reporting the rate of MI and CD, we were therefore unable to conduct subgroup analyses of these outcomes of interest in the present study. Fourth, with regard to the methods, it would have been more appropriate to include a negative control group (patients without ST after PCI) and compare it with results of the EST group and the LST/VLST group. However, only one study was enrolled that established a negative control group [13], therefore, such overall comparison was not possible. Fifth, we included studies using various types of stents (BMS and DES, first-generation DES and second-generation DES, polymer stent and polymer-free stents, etc.) during the index procedure, but meaningful subgroup analysis according to the initial types of stents could not be performed due to the insufficient data of the original articles. Finally, limited data regarding the strategy of antiplatelet use after ST also hampered our ability to explore the effects of this important measure for outcomes of EST and LST/VLST.

## Conclusions

After PCI treatment, patients with EST have worse clinical outcomes in both short- and long-term follow-up than patients with LST/VLST. Treatment for EST patients remains challenging, and further studies are needed that concentrate on determining the optimal treatment strategies for EST.

## Electronic supplementary material

Below is the link to the electronic supplementary material.

Supplementary file1 (DOCX 3316 kb)
