# Supplementary material for: Clinical outcomes after percutaneous coronary intervention for early versus late and very late stent thrombosis: a systematic review and meta-analysis
Source: J Thromb Thrombolysis. 2020 Jul 20;51(3):682–92. doi: 10.1007/s11239-020-02184-7 (PMC8049931; doi:10.1007/s11239-020-02184-7)
Supplement: Supplementary file 1 — Supplementary file1 (DOCX 3316 kb) [file 11239_2020_2184_MOESM1_ESM.docx]

**Supplemental Table 1** Quality assessment of the included studies

| Study | 1 | 2 | 3 | 4 | 5 | 6 | 7 | 8 | Total |
| --- | --- | --- | --- | --- | --- | --- | --- | --- | --- |
| Lemesle **^[1]^** | A | A | A | A | A | B | A | D | 7 |
| Margolis **^[2]^** | A | A | A | B | A | B | A | D | 6 |
| Jones **^[3]^** | A | A | A | B | A | B | A | C | 6 |
| Kimura **^[4]^** | A | A | A | A | A | B | A | D | 7 |
| Kubo **^[5]^** | A | A | A | B | A | B | A | A | 7 |
| Armstrong **^[6]^** | A | A | A | A | A | B | A | A | 8 |
| de la TH **^[7]^** | A | A | A | A | A | B | A | D | 7 |
| Daemen **^[8^]** | A | A | A | A | A | B | A | D | 7 |
| Singh **^[9]^** | A | A | A | A | A | B | A | D | 7 |
| Kuramitsu **^[10]^** | A | A | A | B | A | B | A | B | 7 |
| Mahmoud **^[11]^** | A | A | A | B | A | B | A | D | 6 |
| Lempereur **^[12]^** | A | A | A | B | A | B | A | D | 6 |
| Kim **^[13]^** | A | A | A | B | A | B | A | D | 6 |
| Armstrong **^[14]^** | A | A | A | A | A | B | A | D | 7 |
| Almalla **^[15]^** | A | A | A | A | A | B | A | B | 8 |
| Van Werkum **^[16]^** | A | A | A | A | A | B | A | B | 8 |
| Katsikis **^[17]^** | A | A | A | A | A | B | A | D | 7 |
| Yeo **^[18]^** | A | A | A | A | A | B | A | D | 7 |
| Konishi **^[19]^** | A | A | A | B | A | B | A | A | 7 |
| Tovar Forero **^[20]^** | A | A | A | A | A | B | A | A | 8 |
| Feldman **^[21]^** | A | A | A | A | A | B | A | A | 8 |
| Shimotakahara **^[22]^** | A | A | A | A | A | B | A | D | 7 |
| Kukreja **^[23]^** | A | A | A | A | A | B | A | D | 7 |

1. representativeness of the exposed cohort

2. selection of the non-exposed cohort

3. ascertainment of exposure

4. outcome of interest was not present at start of study

5. comparability of cohorts

6. assessment of outcome

7. long enough follow up

8. adequacy of follow up;

A:1 score; B:0/1 score C:0 score; D:0 score

**Supplemental Table 2** Incidence of MACE at short-term and long-term follow-up

| Study | MACE definition | INH MACE | 30-day MACE | 1-year MACE | Long-term MACE |
| --- | --- | --- | --- | --- | --- |
|  |  | EST vs LST (%) | EST vs LST (%) | EST vs LST (%) | EST vs LST (%) |
| Lemesle **^[1]^** | death, MI, RST | 41.2 vs 20.0 | 47.0 vs 25.0 | 52.9 vs 30.0 | NA |
| Jones **^[3]^** | death, MI, TVR | NA | NA | NA | 46.0 vs 26.8 |
| Kubo **^[5]^** | CD, MI, RST, TLR | NA | NA | 55.5 vs 25.8 | 66.6 vs 38.1 |
| Lempereur **^[12]^** | death, TVR | NA | 30.6 vs 9.2 | 44.4 vs 18.5 | NA |
| Kim **^[13]^** | CD, RST | NA | NA | 30.9 vs 17.3 | NA |
| Almalla **^[15]^** | death, MI, TLR | NA | NA | NA | 43.2 vs 40.0 |
| Van Werkum **^[16]^** | CD, RST | NA | NA | NA | 24.9 vs 28.1 |
| Yeo **^[18]^** | death, MI, stroke | NA | NA | NA | 31.9 vs 34.8 |

MACE: major adverse cardiovascular event; INH: in-hospital; TVR: target vessel revascularization; NA: not available;

TLR: target lesion revascularization; CD: cardiac death; MI: myocardial infarction; RST: recurrent stent thrombosis;

EST: early stent thrombosis; LST: late stent thrombosis (including very late stent thrombosis here); VS: versus


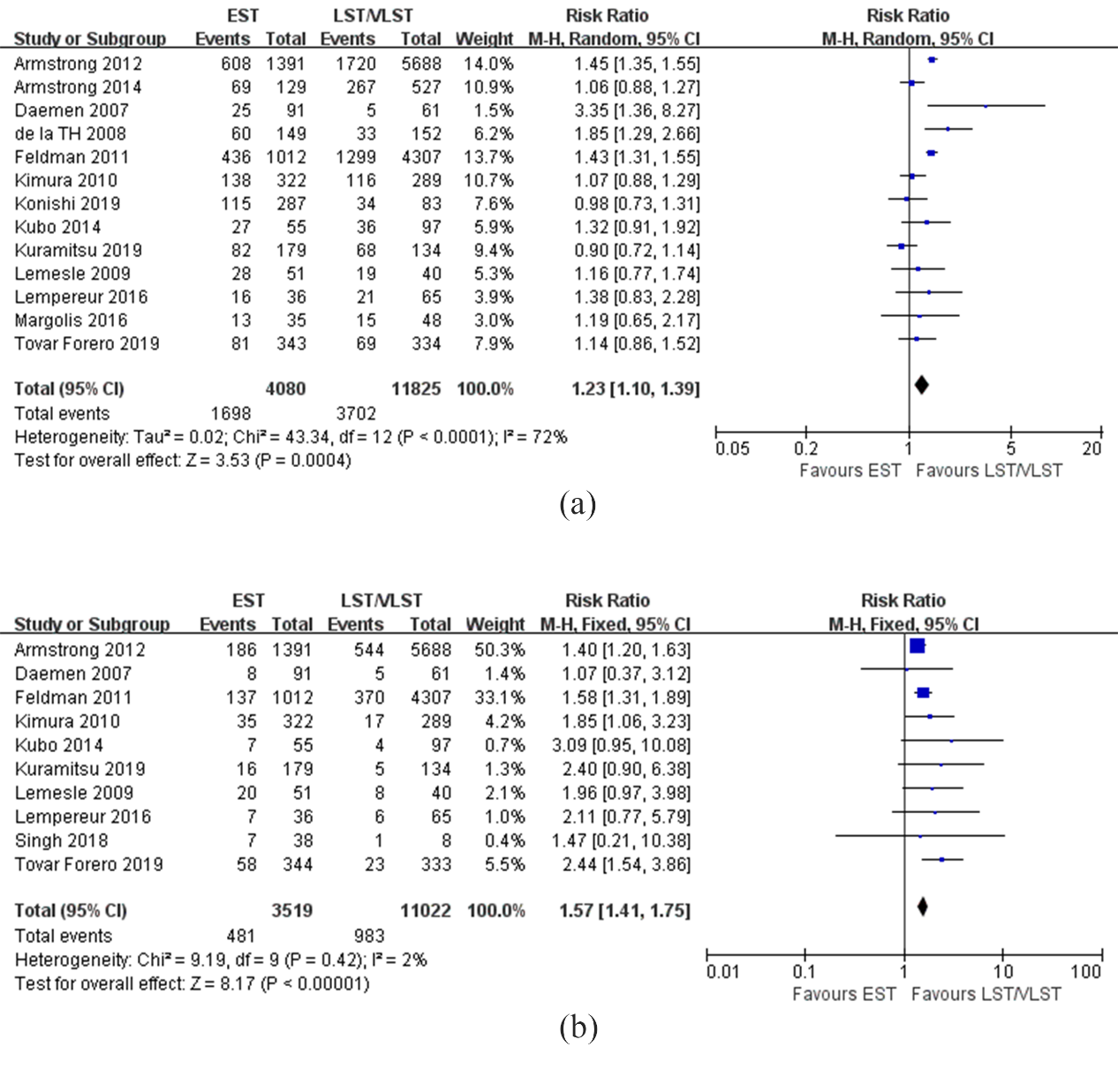


**Supplemental Fig.1** Forest plot with RR for EST vs LST/VLST (a) diabetes mellitus: 41.6% vs 31.3% (b) cardiogenic shock at the time of ST: 13.7% vs 8.9%


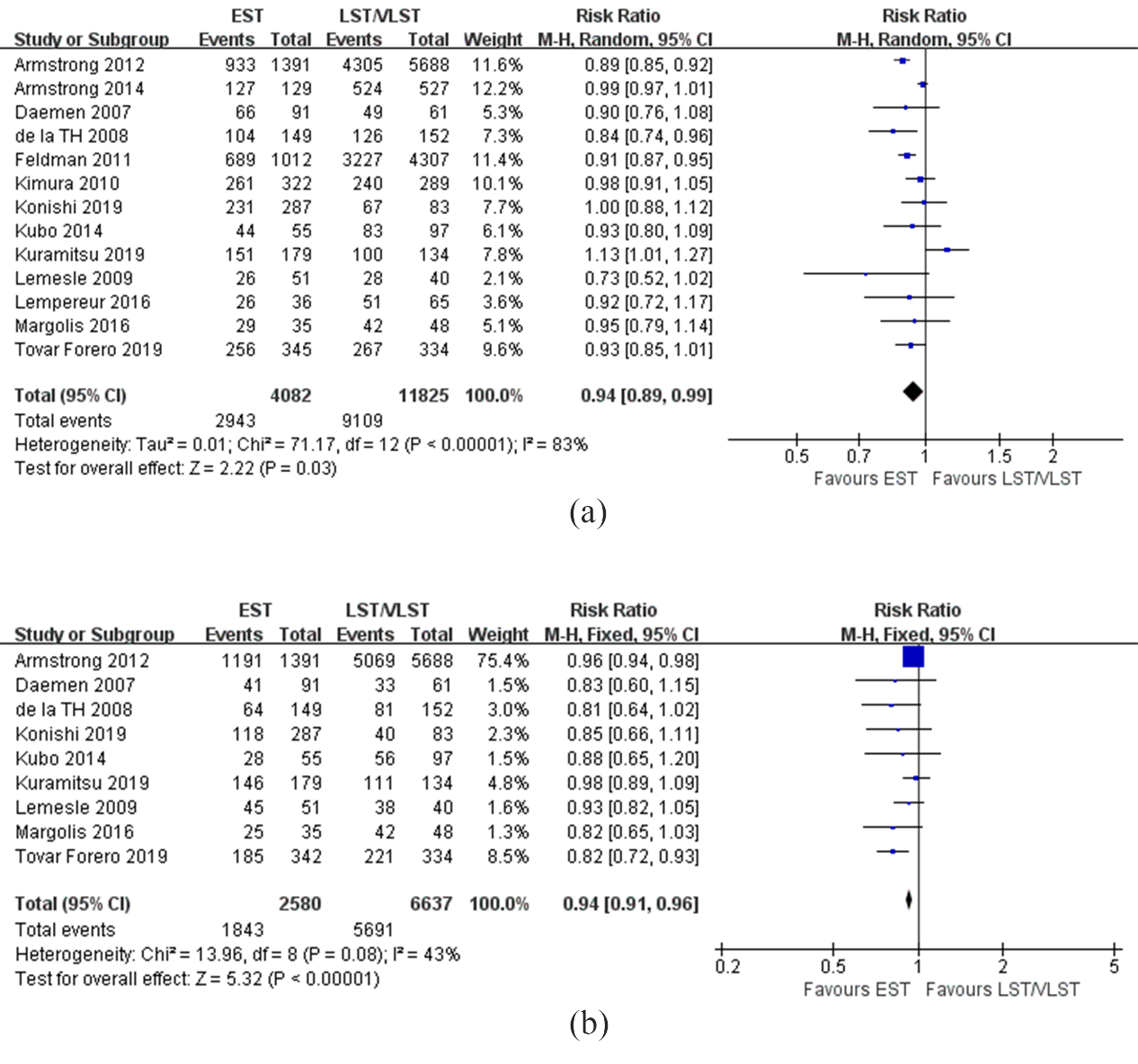


**Supplemental Fig. 2** Forest plot with RR for EST vs LST/VLST (a) male gender: 72.1% vs 77.0% (b) hyperlipemia: 71.4% vs 85.7%

**
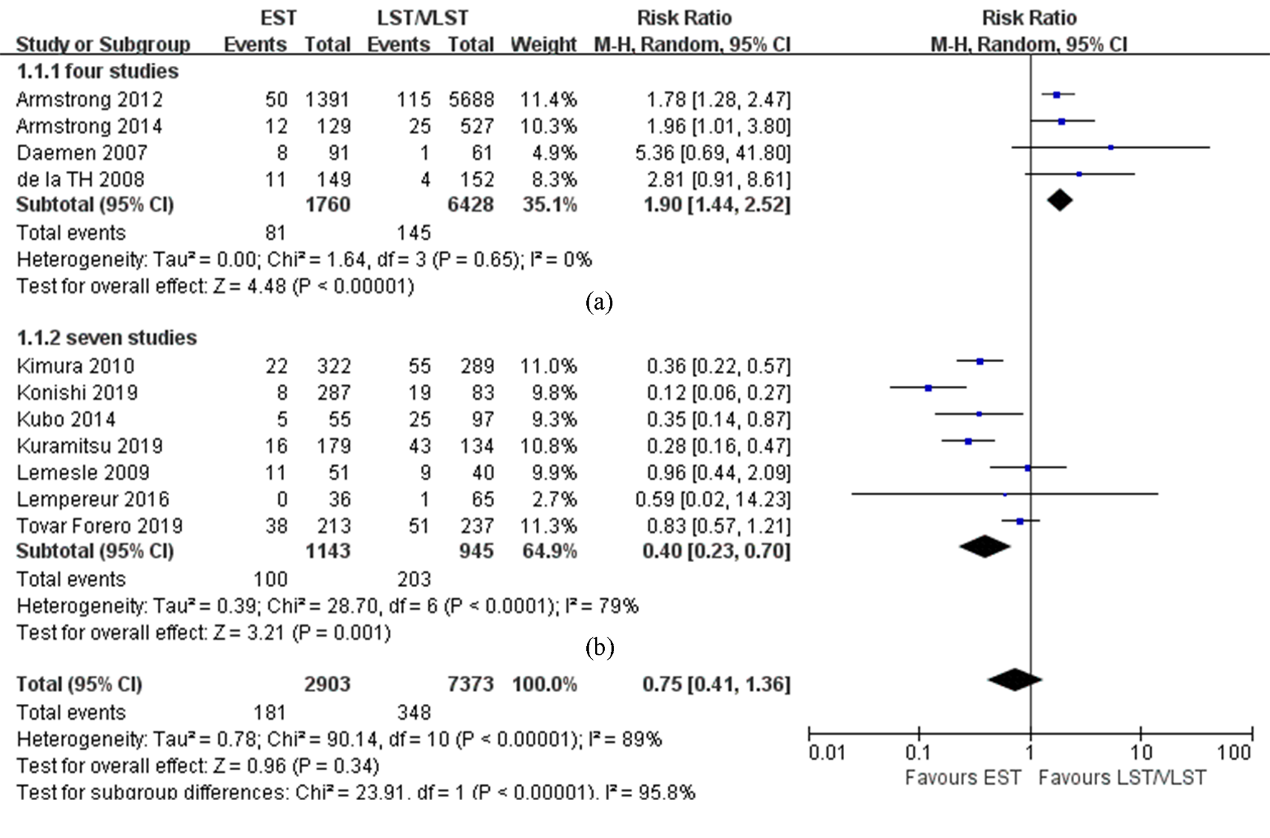
Supplemental Fig. 3** Forest plot with RR for EST vs LST/VLST (a) CKD: 4.6 % vs 2.3 % (b) CKD: 8.7 % vs 21.5 %


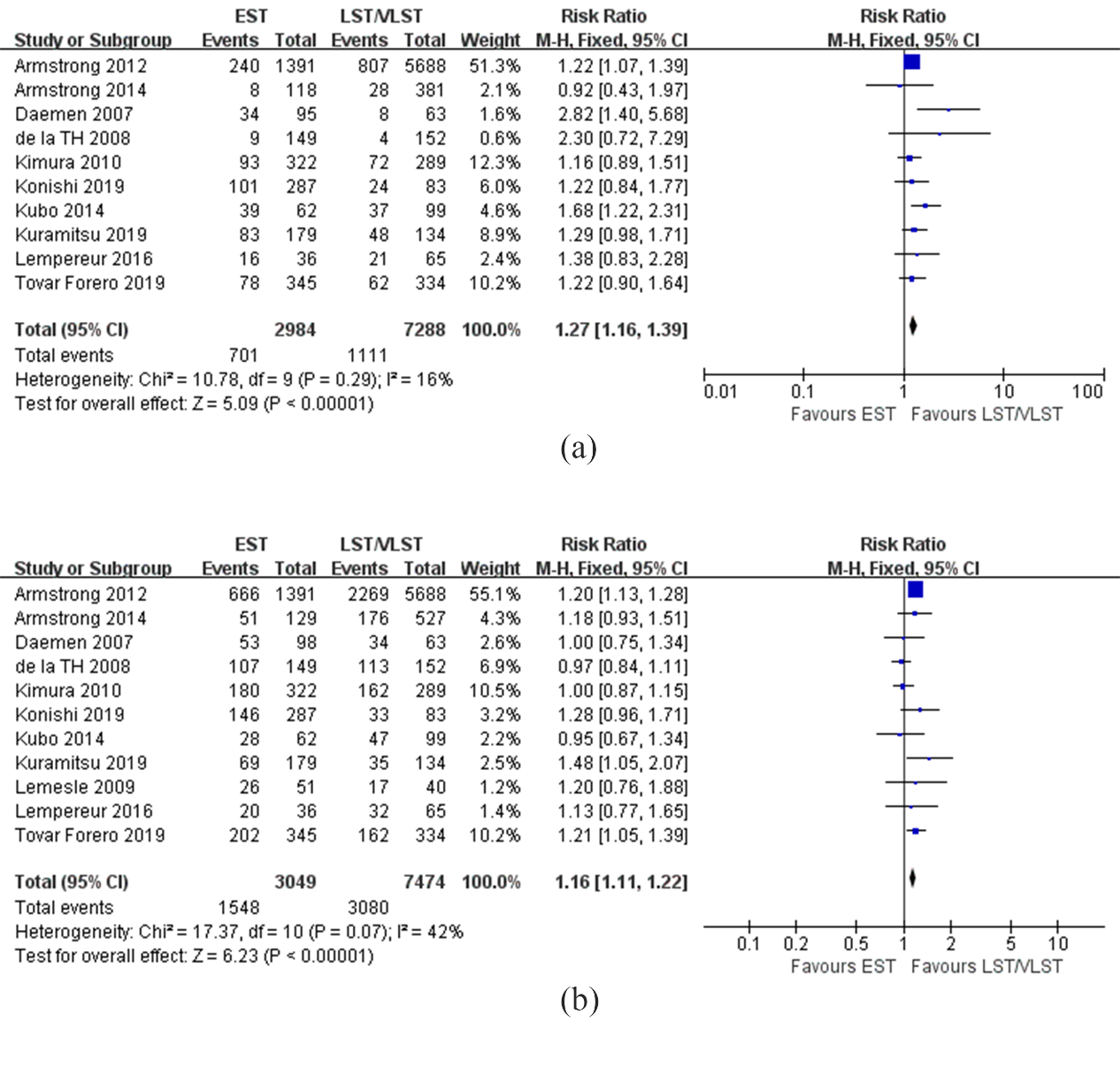


**Supplemental Fig. 4** Forest plot with RR for EST vs LST/VLST (a) bifurcation lesions: 23.5% vs 15.2% (b) left anterior descending artery lesions: 50.8% vs 41.2%


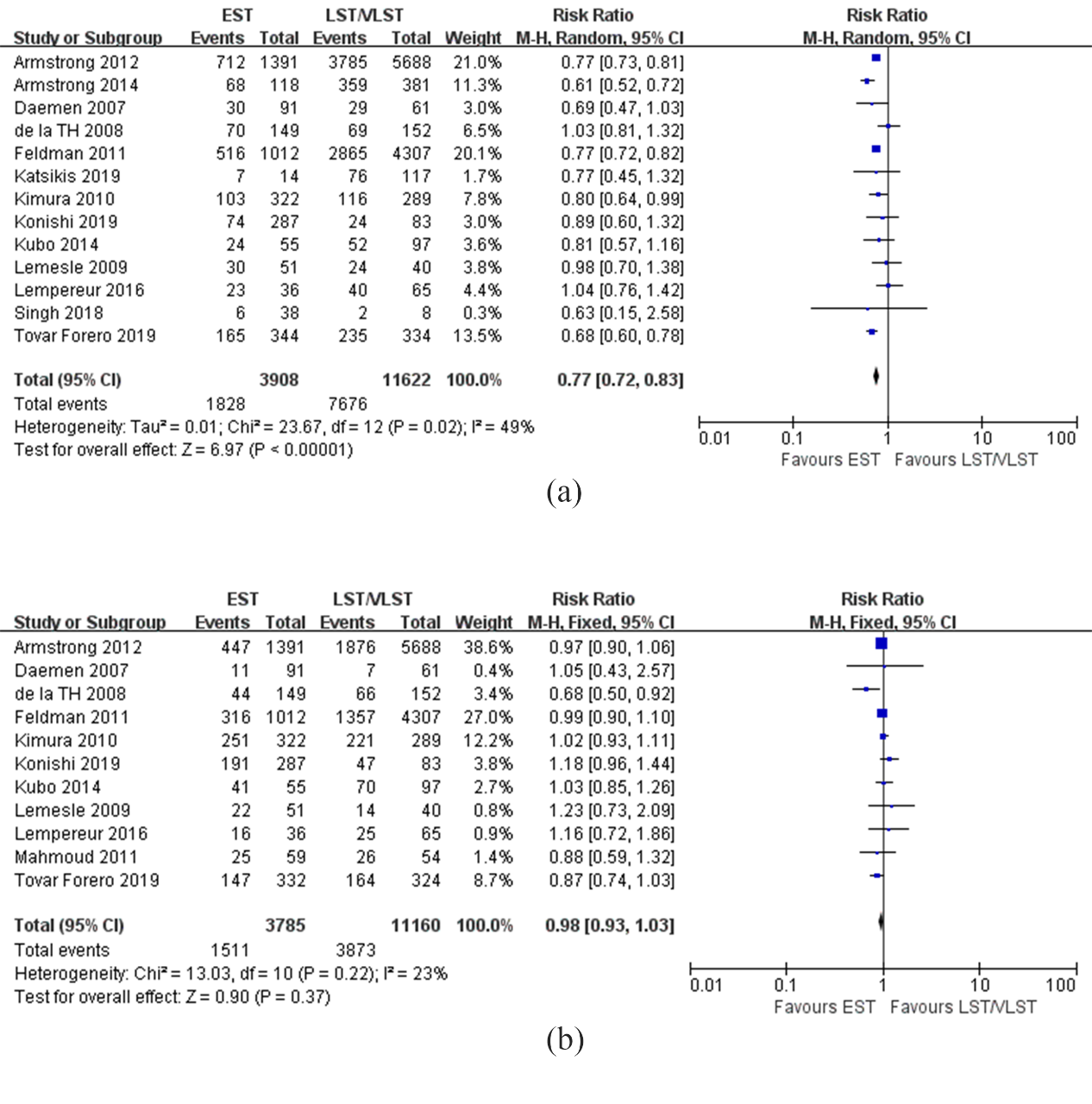


**Supplemental Fig. 5** Forest plot with RR for EST vs LST/VLST (a) additional stent: 46.8% vs 66.0% (b) thrombus aspiration: 30.4% vs 34.7%


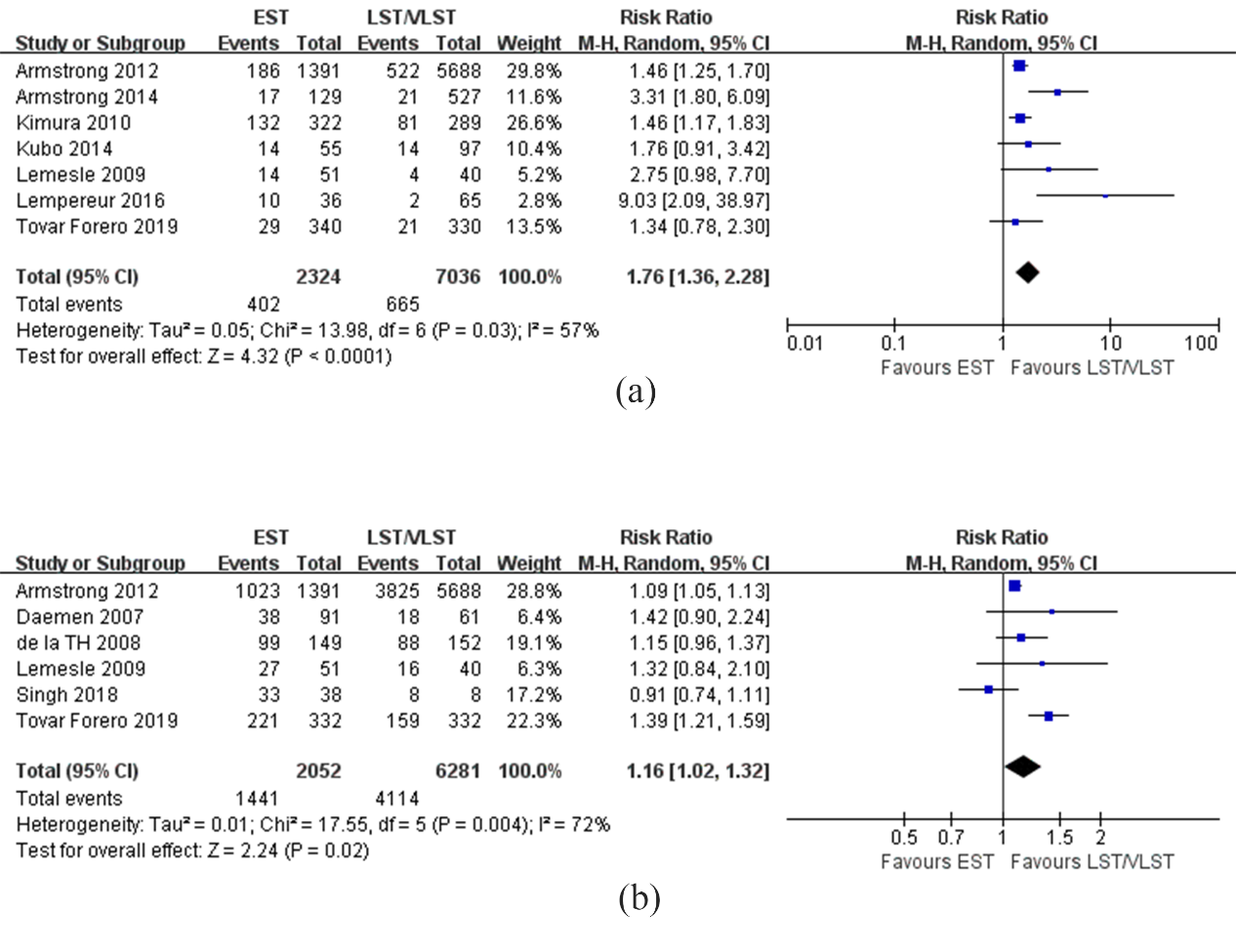


**Supplemental Fig. 6** Forest plot with RR for EST vs LST/VLST (a) intra-aortic balloon pump: 17.3% vs 9.5% (b) glycoprotein IIb/IIIa inhibitors: 70.2% vs 65.5%


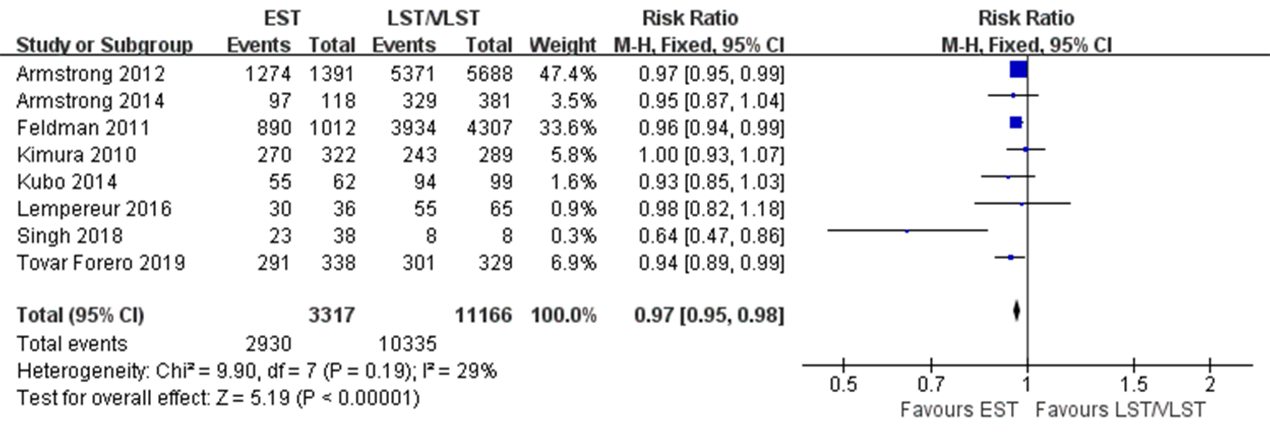


**Supplemental Fig. 7** Forest plot with RR for EST vs LST/VLST: post-PCI TIMI flow grade 3: 88.3% vs 92.6%


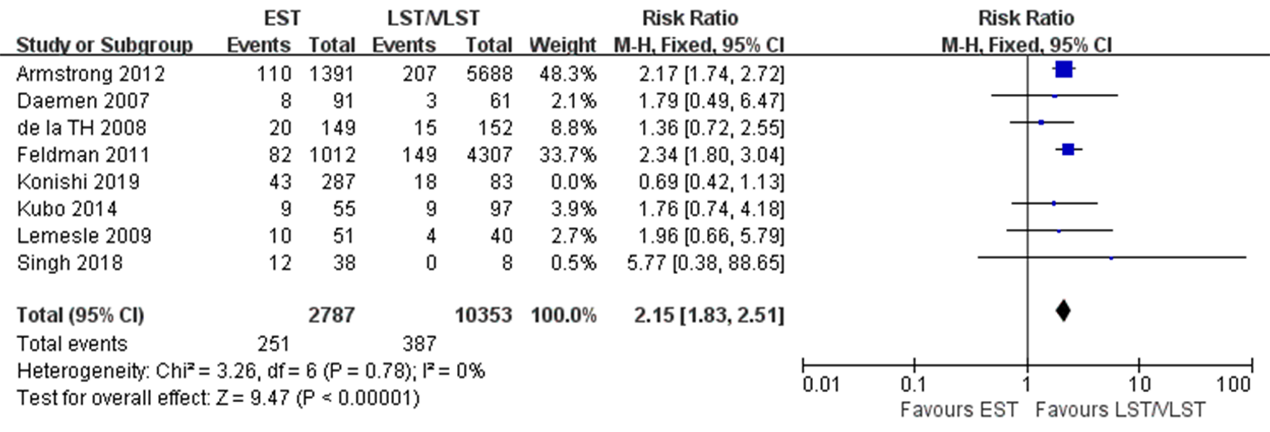


Supplement**al Fig. 8** Forest plot with RR for EST vs LST/VLST: in-hospital mortality (removing the Konishi study): 9.0% vs 3.7%
